# Donor noradrenaline use is associated with better allograft survival in recipients of pancreas transplantation

**DOI:** 10.1308/rcsann.2022.0161

**Published:** 2023-03-16

**Authors:** IM Shapey, A Summers, P Yiannoullou, C Fullwood, T Augustine, MK Rutter, D van Dellen

**Affiliations:** ^1^ University of Manchester, UK; ^2^ Manchester University NHS Foundation Trust, UK

**Keywords:** Pancreas, Transplant, Noradrenaline, Organ donor

## Abstract

**Introduction:**

Outcomes following pancreas transplantation are suboptimal and better donor selection is required to improve this. Vasoactive drugs (VaD) are commonly used to correct the abnormal haemodynamics of organ donors in intensive care units. VaDs can differentially affect insulin secretion positively (dobutamine) or negatively (noradrenaline). The hypothesis was that some VaDs might induce beta-cell stress or rest and therefore impact pancreas transplant outcomes. The aim of the study was to assess relationships between VaD use and pancreas transplant graft survival.

**Methods:**

Data from the UK Transplant Registry on all pancreas transplants performed between 2004 and 2016 with complete follow-up data were included. Univariable- and multivariable-adjusted Cox regression analyses determined risks of graft failure associated with VaD use.

**Results:**

In 2,183 pancreas transplants, VaDs were used in the following numbers of donors: dobutamine 76 (3.5%), dopamine 84 (3.8%), adrenaline 161 (7.4%), noradrenaline 1,589 (72.8%) and vasopressin 1,219 (55.8%). In multivariable models, adjusted for covariates and the co-administration of other VaDs, noradrenaline use (vs non-use) was a strong predictor of better graft survival (hazard ratio [95% confidence interval] 0.77 [0.64–0.94], *p* = 0.01).

**Conclusions:**

Noradrenaline use was associated with better graft survival in models adjusted for donor and recipient variables – this may be related to inhibition of pancreatic insulin secretion initiating pancreatic beta-cell ‘rest’. Further research is required to replicate these findings and establish whether relationships are causal. Identification of alternative methods of inducing beta-cell rest could be valuable in improving graft outcomes.

## Introduction

Pancreas transplantation is a highly effective life-saving therapy for patients with insulin-dependent diabetes and end-organ damage from renal failure or unavoidable episodes of severe hypoglycaemia. Owing to a well-documented shortage of organs for transplantation, there is a need to optimise methods for the assessment and selection of high-quality pancreases. Outcomes are improving, but historically remain far from ideal: six of ten pancreas grafts have failed at 5 years when transplanted alone, and three of ten have failed at 5 years when the pancreas is transplanted together with a kidney.^1^ Technical considerations provide one explanation for these poorer outcomes and these have evolved and improved considerably over time.^2^ Immunological factors including sensitisation from prior kidney transplants is a probable factor behind the discrepancy in outcomes between synchronous pancreases and kidney transplants compared with pancreases transplanted after kidney transplantation.^3^

In organ donors, the process of brainstem death results in high levels of systemic catecholamines affecting all organs, including the pancreas.^4,5^ Catecholamine-based vasoactive drugs (VaDs) are commonly used to correct the hypotension and reduced cardiac output experienced by organ donors in intensive care units. However, this may inadvertently affect organ quality. The donor care bundle produced by NHS Blood and Transplant recommends aiming to achieve mean arterial pressure values between 60 and 80mmHg by correction of hypovolaemia, with the administration of fluid boluses in the first instance and then vasopressors.^6^ Vasopressin is recommended as the vasopressor of choice, and it is advised to wean or stop catecholamines. If inotropes are to be used then dopamine is recommended as the agent of choice, although dobutamine is often used.

Pancreatic beta cells receive sympathetic autonomic innervation and express α2-adrenergic receptors that inhibit insulin secretion and β2-adrenergic receptors that stimulate insulin secretion.^7,8^ Adrenergic receptors are G protein-coupled receptors (GPCRs). VaDs vary in their affinity for certain GPCRs and as such may have variable impacts on insulin secretion (Table 1).

**Table 1:** Effect of vasoactive drugs on insulin and glucagon secretion

Vasoactive drug	Receptor affinity	Affinity	Net effect on glycaemic control
Noradrenaline	α2 adrenergic β2 adrenergic	++++ +	Inhibits insulin secretion Stimulates glucagon secretion
Adrenaline	α2 adrenergic β2 adrenergic	+ +++	Stimulates insulin secretion
Vasopressin	V1a V1b	+++ +++	Simulates both insulin and glucagon secretion
Dobutamine	α1/2 adrenergic β1 adrenergic β2 adrenergic	+ (−ve isomer) − (+ve isomer) ++++ +	Stimulates insulin secretion Stimulates glucagon secretion
Dopamine	D2-like (D2/D3) α2 adrenergic β2 adrenergic	++++ + +	Inhibits insulin secretion Stimulates glucagon secretion

− = negative; + = weak; ++ = moderate; +++ = strong; ++++ = very strong

The relationship between VaDs and outcomes after pancreas transplantation is unknown. Because these drugs have effects on insulin secretion, we hypothesised that some VaDs might impact pancreas transplant outcomes. This study aimed to assess relationships of VaD use to pancreas transplant graft survival.

## Methods

Data on all pancreas transplants performed between 1 January 2004 and 1 November 2016 from the UK Transplant Registry, held by NHS Blood and Transplant, were reviewed. All data were collected prospectively with written informed consent from donor families and transplant recipients.

All-cause pancreas graft failure was defined as the time from transplant to loss of insulin independence. Patients dying with a functioning graft were censored.

The distributions of all donor and recipient variables were assessed for form and completeness. Cases with missing outcome data (*n* = 88) were excluded. Data for cold ischaemic time were missing in 9% of cases, donor creatinine in 12% of cases and recipient body mass index (BMI) in 27% of cases – this was addressed by using pooled results from multiple imputation using the fully conditional specification (Markov Chain Monte Carlo) algorithm. Missing data for all other variables were infrequent (< 1%) and cases with missing data relating to independent variables were excluded from individual analyses at that level.

### Statistical analyses

We assessed relationships between exposures (VaDs) and donor or recipient clinical variables to identify potential confounders of relationships between exposures (VaDs) and outcome (graft failure) using Fisher’s exact test, Student’s *t*-test or the Mann–Whitney *U*-test as appropriate. Univariable Cox regression determined hazard ratios (HR [95% confidence interval {CI}]) for graft failure associated with clinical variables identified as being related to exposures to increase understanding of their potential role as confounders (*p *< 0.1). The association between VaD use and graft failure was determined using a Cox regression model, which was adjusted for co-administration of VaDs and also clinical variables related to graft failure in a Cox regression model (*p *< 0.05).

Statistical analyses were performed using IBM SPSS statistics version 22 (IBM, Armonk, NY).

## Results

Some 2,271 pancreas transplants were performed in the UK during the study period, of which 88 (3.9%) were excluded because of missing survival data, leaving 2,183 cases in the analysis. Simultaneous pancreas and kidney (SPK) transplantation comprised 1,879 (86.1%) of complete cases, pancreas after kidney (PAK) transplantation accounted for 153 (7.0%) cases and pancreas transplantation alone (PTA) accounted for 151 (6.9%). All-cause pancreas graft failures totalled 525 (24.0%) over a median (interquartile range) follow-up period of 4 (1–8) years.

VaDs were used in the following numbers (proportions) of donors: dobutamine 76 (3.5%), dopamine 84 (3.8%), adrenaline 161 (7.4%), noradrenaline 1,589 (72.8%) and vasopressin 1,219 (55.8%). No VaDs were received by 359 (16.4%) donors, whereas 737 (33.8%) received one VaD, 1,023 (46.9%) received two, 141 (6.5%) received three, 13 (0.6%) received four and 1 (< 0.1%) donor received all five VaDs. In all patients receiving inotropes (dobutamine or dopamine), vasopressors (noradrenaline, adrenaline or vasopressin) were also co-administered. Noradrenaline was co-administered in 67 (88.2%) donors receiving dobutamine and in 55 (65.5%) donors receiving dopamine.

Use of noradrenaline in donors was associated with both donor and recipient non-White ethnicity, previous history of hypertension, previous history of cardiac disease, peri-retrieval hypotension, donation after brain death (DBD), trauma and hypoxic brain injury as cause of death, higher donor creatinine and amylase values, and greater use of vasopressin and insulin (Tables 2 and 3).

**Table 2:** Donor and recipient characteristics associated with noradrenaline use in pancreas transplant donors

Donor and recipient variables	Donor noradrenaline use		*p*-value
	No (*n *= 594)	Yes (*n *= 1,589)	
Donor variables			
Age*	36 (13.7)	35 (13.4)	0.083
Sex (male)	293 (48)	845 (51)	0.171
Ethnicity (White)	576 (96%)	1523 (93)	0.036
BMI*	24 (4)	24 (4)	0.542
Smoking	304 (50)	830 (51)	0.740
Alcohol excess	53 (9)	113 (8)	0.121
Past hypertension	69 (11)	125 (8)	**0.007**
Past cardiac disease	25 (4)	41 (3)	**0.048**
Cardiac arrest	195 (32)	479 (29)	0.195
Peri-retrieval hypotension	333 (5)	1,082 (67)	**< 0.001**
Donor type (DBD)	451 (74)	1,427 (86)	**< 0.001**
Cause of death			
Trauma	82 (13)	292 (18)	**0** **.** **014**
Meningitis	15 (2)	39 (2)	0.92
Stroke (thromboembolic)	36 (6)	88 (5)	0.61
Intracranial haemorrhage	296 (48)	843 (51)	0.254
Hypoxic brain damage	140 (23)	281 (17)	**< 0** **.** **001**
Brain tumour	13 (2)	22 (1)	0.175
Other	32 (5)	91 (6)	0.791
Creatinine*	76 (42)	83 (50)	**0.003**
Creatinine > 2.5mg/dl	9 (2)	38 (2)	0.397
Amylase, IU/L†	57 (36–100)	64 (38–121)	**0.004**
CIT	12 (3)	12 (3)	0.598
HLA group			
1	4 (<1)	10 (<1)	0.772
2	39 (7)	95 (6)	n.a.
3	170 (29)	497 (31)	n.a.
4	373 (63)	995 (63)	n.a.
Donor vasoactive drug use			
None	338 (57)	n.a.	n.a.
Noradrenaline	n.a.	n.a.	n.a.
Adrenaline	50 (8)	111 (7)	0.23
Dobutamine	9 (2)	67 (4)	0.002
Dopamine	31 (5)	55 (3)	0.062
Vasopressin	213 (36)	1,011 (64)	**< 0** **.** **00001**
Insulin	222 (37)	900 (57)	**< 0** **.** **00001**
Triiodothyronine	139 (24)	542 (34)	**< 0** **.** **00001**
Methylprednisolone	195 (33)	598 (37)	0.079
Recipient variables			
Age*	42 (8)	42 (8)	0.147
Sex (male)	338 (57)	912 (57)	0.845
Ethnicity (White)	541 (91)	1,422 (89)	0.039
BMI*	25 (4)	25 (5)	0.204
Transplant type (SPK)	492 (83)	1,387 (87)	0.094

BMI = body mass index; CIT = cold ischaemic time; DBD = donation after brain death; HLA = human leucocyte antigen; n.a. = not applicable; SPK = simultaneous pancreas–kidney. Alcohol excess = ≥ 7units/day; cardiac arrest = cessation of circulation during the acute event that led to organ donation; cardiac disease = either ischaemic heart disease of valvular disease; donor drug use = presented as use vs non-use; methylprednisolone use = 15mg/kg to a maximum of 1g as outlined in the donor care bundle (data on total prescribed dosage for other drugs were not available); smoking = either past or present

*Age (years), BMI (kg/m^2^) and creatinine μmol/L) are continuous data presented as mean ± SD. Missing data were handled by multiple imputation (creatinine, 12%; CIT, 9%; recipient BMI, 27% of cases).

†Amylase is continuous data presented as median (interquartile range).

All binary data are presented as *n* (%).

All available variables were included in analysis.

**Table 3:** HLA mismatch groups

Level	HLA mismatch summary	HLA mismatch combinations included
1	0	0
2	[0 DR and 0/1 B]	100, 010, 110, 200, 210
3	[0 DR and 2 B] or [1 DR and 0/1 B]	020, 120, 220, 001, 101, 201, 011, 111, 211
4	[1 DR and 2 B] or [2 DR]	021, 121, 221, 002, 102, 202, 012, 112, 212, 022, 122, 222

In a univariable Cox regression model, use of noradrenaline was related to a lower risk of pancreas graft failure (HR [95% CI] 0.74 [0.61–0.89], *p* = 0.001) (Table 4). In the same analysis the following were related (*p *< 0.1) to all-cause pancreas graft failure: increasing donor age; increasing donor BMI; donor history of previous hypertension; cardiac arrest (but not its duration) during the donation phase; donor cause of death involving trauma, intracranial haemorrhage and hypoxic brain injury; increasing recipient age; type of transplant; and increasing cold ischaemic time (Table 4). SPK transplantation was associated with superior graft survival compared with PTA/PAK (HR [95% CI] for graft survival 0.38 [0.31–0.46], *p *< 0.001), whereas transplantation of solitary pancreas grafts, either as PTA (2.13 [1.63–2.77], *p *< 0.001) or PAK (2.63 [2.07–3.34], *p *< 0.001), was associated with poorer graft survival.

**Table 4:** Donor and recipient factors predicting risk of graft failure after pancreas transplantation

Predictor variables	*N*	HR	95% CI	*p*-value
Donor variables				
Age*	35 (13)	1.02	1.01–1.02	**< 0** **.** **001**
Sex (male)*	1,138 (52)	1.05	0.89–1.25	0.57
Ethnicity (White)	2,099 (96)	1.13	0.77–1.65	0.53
BMI, kg/m^2^*	24 (4)	1.03	1.00–1.05	**0** **.** **03**
Smoking	1,134 (52)	1	0.84–1.12	0.99
Alcohol excess	166 (8)	1.26	0.94–1.70	0.13
** **Past hypertension	194 (9)	1.33	1.00–1.77	**0** **.** **05**
Past cardiac disease	65 (3)	1.04	0.64–1.69	0.87
Cardiac arrest	674 (31)	0.82	0.67–1.01	**0** **.** **06**
Peri-retrieval hypotension	1,415 (65)	1.04	0.87–1.26	0.65
Donor type (DBD)	1,878 (86)	0.85	0.67–1.07	0.16
Cause of death				
Trauma	374 (17)	0.79	0.63–1.00	**0** **.** **05**
Meningitis	54 (2)	0.65	0.34–1.26	0.20
Stroke (thromboembolic)	124 (6)	1.78	0.82–1.68	0.38
Intracranial haemorrhage	1,139 (52)	1.26	1.06–1.50	**0** **.** **01**
Hypoxic brain damage	421 (19)	0.76	0.59–0.99	**0** **.** **04**
Brain tumour	35 (2)	1.05	0.52–2.10	0.90
Other	126 (6)	1.17	0.82–1.66	0.39
Creatinine*	82 (48)	1	1.00–1.00	0.63
Creatinine >2.5mg/dl (221μmol/L)	47 (2)	1.31	0.49–3.48	0.54
Amylase†	61 (37–115)	1	1.0–1.0	0.21
Donor vasoactive drug use				
None	359 (16)	1.29	1.03–1.61	**0** **.** **03**
Noradrenaline	1,589 (73)	0.74	0.61–0.89	**0** **.** **001**
Adrenaline	161 (7)	0.79	0.55–1.12	0.18
Dopamine	76 (4)	1.13	0.75–1.70	0.57
Dobutamine	86 (4)	1.42	0.94–2.13	**0** **.** **10**
Vasopressin	1,224 (56)	0.94	0.79–1.12	0.49
Insulin	1,122 (51)	0.95	0.8–1.13	0.59
Methylprednisolone	681 (31)	1.05	0.87–1.26	0.60
Triiodothyronine	793 (36)	1.1	0.92–1.32	0.30
Recipient variables				
Age*	42 (8)	0.99	0.98–1.00	0.04
Sex (male)	1,250 (57)	1.03	0.87–1.22	0.74
Ethnicity (white)	1,963 (90)	1.11	0.81–1.51	0.52
BMI*	25 (4)	1.02	0.99–1.05	0.11
Transplant type (SPK)	1,879 (86)	0.38	0.31–0.46	**< 0** **.** **00001**
CIT*	12 (3)	1.07	1.05–1.10	**< 0** **.** **001**
HLA group				
1	14 (<1)	1.52	0.63–3.68	0.35
2	134 (6)	0.65	0.43–0.98	**0** **.** **04**
3	667 (31)	1.08	0.90–1.30	0.40
4	1,368 (63)	1.01	0.85–1.21	0.88

BMI = body mass index; CI = confidence interval; CIT = cold ischaemic time; DBD = donation after brain death; HLA = human leucocyte antigen; HR = hazard ratio; SPK = simultaneous pancreas–kidney. Alcohol excess = ≥7 units/day; cardiac arrest = cessation of circulation during the acute event that led to organ donation; cardiac disease = either ischaemic heart disease of valvular disease; donor drug use = presented as use vs non-use; methylprednisolone use = 15mg/kg to a maximum of 1g as outlined in the donor care bundle (data on total prescribed dosage for other drugs were not available); smoking = either past or present

*Age (years), BMI (kg/m^2^), creatinine (μmol/L) and CIT are continuous data presented as mean ± SD. Missing data were handled by multiple imputation (creatinine, 12%; CIT, 9%; recipient BMI, 27% of cases).

†Amylase is continuous data presented as median (IQR).

All binary data are presented as *n* (%).

All available variables were included in analysis

Use of donor noradrenaline was not related to patient survival (HR [95% CI] 0.94 [0.72–1.23], *p* = 0.666), nor was duration of cardiac arrest.

**Figure 1:**
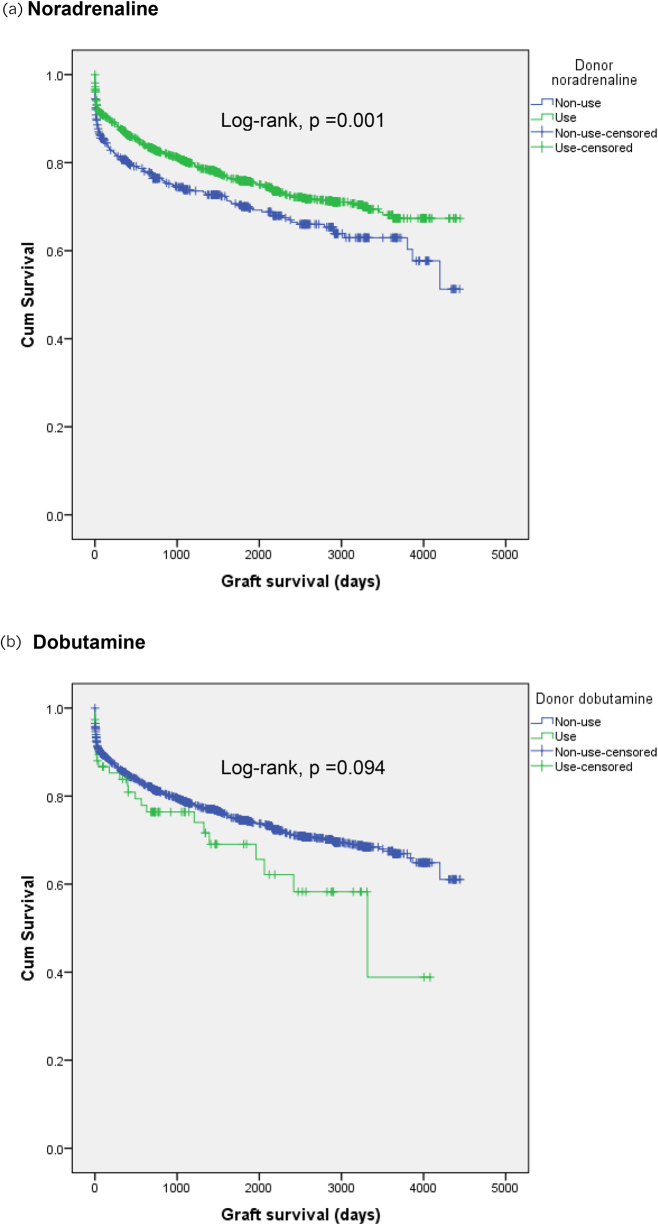
Kaplan–Meier curves showing the survival probability after pancreas transplantation stratified by vasoactive drug use: (a) noradrenaline, (b) dobutamine

After adjusting for co-administration and non-administration of donor drugs, only noradrenaline use was associated with better graft survival (HR [95% CI] 0.70 (0.53–0.92), *p* = 0.01) although dobutamine use was associated with a non-significant trend for adverse graft survival (1.50 [0.99–2.27], *p* = 0.055) (Table 5). After including all potential confounders in a multivariable-adjusted Cox regression model, donor noradrenaline use was significantly related to a 26% lower risk of pancreas graft loss (HR [95% CI] 0.77 [0.64–0.94], *p *= 0.01) (Table 6; Figure 1).

**Table 5:** Donor vasoactive drug use adjusted for co-administration and non-administration predicting risk of graft failure after pancreas transplantation (*n* = 2,183)

Donor drug use	HR	95% CI	*p*-value
None	0.94	0.64–1.36	0.72
Noradrenaline	0.70	0.53–0.92	**0** **.** **01**
Adrenaline	0.75	0.52–1.09	0.13
Dopamine	1.06	0.69–1.61	0.80
Dobutamine	1.50	0.99–2.27	**0** **.** **06**
Vasopressin	0.94	0.76–1.16	0.56
Insulin	0.98	0.82–1.18	0.85
Triiodothyronine	1.15	0.94–1.39	0.17
Methylprednisolone	1.04	1.04–0.86	0.66

CI = confidence interval; HR = hazard ratio

**Table 6:** Donor and recipient factors predicting risk of graft failure after pancreas transplantation (*n *= 2,183)

Variables	HR	95% CI	*p*-value
Donor variables			
Age	1.02	1.01–1.02	< 0.001
BMI	1.02	0.99–1.05	0.22
Hypertension	0.98	0.71–1.35	0.88
Cardiac arrest	0.92	0.71–1.20	0.53
Cause of death			
Trauma	0.82	0.62–1.20	0.38
ICH	0.99	0.76–1.29	0.95
HBI	0.82	0.62–1.20	0.31
Noradrenaline use	0.77	0.64–0.94	0.01
Dobutamine use	1.52	0.97–2.39	0.07
Recipient variables			
Age	0.98	0.97–0.99	< 0.01
Transplant type	0.36	0.30–0.45	< 0.00001
CIT	1.08	1.05–1.11	< 0.00001
HLA group 2	0.72	0.48–1.09	0.12

A = BMI = body mass index; CI = confidence interval; CIT = cold ischaemic time; HBI = hypoxic brain injury; HLA = human leucocyte antigen; HR = hazard ratio; ICH = intracranial haemorrhage

Data are hazard ratios (95% CI) from multivariable Cox regression. Variables were included in the model if they were related to graft failure.

HLA group 2 (inclusive of 100, 010, 110, 200, 210 HLA-A, -B and -DR mismatches)

## Discussion

This study demonstrates that donor use of noradrenaline was associated with better pancreas graft survival after pancreas transplantation. Noradrenaline use appeared beneficial irrespective of the type of pancreas transplant (PTA or PAK procedures rather than SPK). If validated, these data have implications for donor selection and the optimal management of pancreas transplant donors.

### Prior studies

Only one previous study investigated the relationship between VaDs and outcome after pancreas transplantation. In this small case series of 59 pancreas transplant recipients, there was no difference in outcomes in relation to the use of catecholamine-based VaDs or desmopressin.^9^ This small study may have been underpowered to detect differences in outcomes in relation to the exposure.

Poorer graft survival in patients receiving PAK remains a topic of on-going interest and debate regarding the factors accounting for this difference. Immunological factors are likely to be key contributors, and our data report that more than 60% of patients undergoing pancreas transplantation were in mismatch group 4 ([1 DR and 2 B] or [2 DR]). The effects of high mismatch in the PAK cohort are likely to compound the effects of prior sensitisation from initial kidney transplantation. These data are corroborated by an analysis of UK Transplant Registry data comparing live donor kidney transplantation with SPK, where only 30% of live donor kidney transplants were in group 4.^10^ In patients with a pancreas graft functioning for more than 90 days, survival was superior to that experienced by recipients of live donor kidney transplantation. These data illustrate the important beneficial effects of good glycaemic control on patient survival. Interestingly, in a single-centre analysis from Spain, pancreas graft survival was worse with live donor kidney transplantation compared with deceased donor transplantation.^3^

### Mechanistic insights

It is unclear how noradrenaline use is associated with better graft outcomes and what underlying mechanisms may contribute to this finding. However, we can speculate that the inhibition of pancreatic insulin secretion leads to a period of beta-cell ‘rest’. Beta-cell rest is an important concept in diabetes medicine, and can lead to improvements in glycaemic control and a reduction in beta-cell death.^11^ This hypothesis is supported by our data: donors who received noradrenaline were significantly more likely to have also required insulin to treat hyperglycaemia.

Noradrenaline has a high affinity for α2 receptors and little effect on β2 receptors. Noradrenaline exerts its potent inhibitory effect on insulin secretion by activating GPCRs in several ways. The predominant mechanism is through inhibition of the exocytosis of secretory granules, also termed the ‘distal’ effect, by its action on G_βγ_ protein.^12,13^ Heterotrimeric Gi proteins (subsets 1 and 2) also act by retarding the process of refilling empty insulin secretory granules, thus disrupting the state of the readily releasable pool of insulin-containing granules.^14^ Go proteins reduce the number of docked granules (i.e. those granules at an immediate state of secretory readiness).^14^ Activation of K_ATP_ channels by Gi/Go proteins hyperpolarises the beta cell and prevents gating of voltage-dependent Ca^2+^ channels, which increase intracellular Ca^2+^ concentration and trigger insulin release.^15^ Adenylyl cyclase, the enzyme that catalyses the conversion of ATP to cyclic-AMP and plays a major role in mediating glucose-stimulated insulin secretion, is also subject to regulation by Gz proteins and hence is inhibited by noradrenaline.^16-18^ Finally, Gz proteins regulate endocytosis of insulin secretory vesicles.^19^

The data in this study were suggestive of a potentially adverse effect of donor dobutamine use on graft survival, although the results were not statistically significant. Dobutamine is a synthetic catecholamine with a complex mixture of affinity to adrenergic receptors. The (–)-isomer of dobutamine is a potent partial alpha agonist, whereas the (+)-isomer is a potent beta agonist and alpha antagonist.^20^ When dobutamine is administered clinically (in its racemic form) it exerts a partial alpha agonist effect and antagonises the alpha effect in physiological circumstances of high sympathetic nervous activity, such as brain death.^21^ It exerts a strong beta-1 activity, but only partial agonism of the beta-2 receptor and competitively inhibits the effect of adrenaline at the beta-2 receptor.^20,22^ Racemic dobutamine is reported to have a higher affinity for the β1-adrenergic receptor than for the β2-adrenergic receptor in a 3:1 ratio.^23^ As such, dobutamine has been shown to stimulate insulin secretion in healthy normal men.^24^

Adrenaline, which is synthesised through the methylation of the primary amine of noradrenaline, has a lower affinity for α2-adrenoceptors (AR) but a greater affinity for β2-receptors.^20^ Its mechanism of action upon the α2-AR in the pancreatic beta cell is the same as for norepinephrine. However, the underlying mechanism of action and mediators of β2-AR-induced insulin secretion are less well understood. Nonetheless, there is a clear causal^21^, and dose-dependent relationship between β2 agonists and insulin secretion from islet cells.^22,23^ Glucagon secretion from pancreatic alpha cells was initially believed to be regulated by β-Ars; however, this evidence was based primarily in non-human subjects, and not supported by data from human studies.^22,24-26^

Dopamine, the most basic catechol structure, is the upstream molecule from which both norepinephrine and epinephrine are derived. Dopamine receptors can be classified into two groups: D1-like, which include D1 and D5 receptors; and D2-like, which include D2, D3 and D4. The presence of D2-like receptors on pancreatic beta cells has been confirmed, and agonism of these receptors inhibits glucose-stimulated insulin secretion via modulation of Gα_i_ receptors.^27,28^ These findings were confirmed by Simpson *et al* who also demonstrated an autocrine role for dopamine in the regulation of insulin secretion.^29^

Vasopressin, meanwhile, via Gq proteins, stimulates both insulin and glucagon secretion.^30,31^ Vasopressin receptors have been identified on both pancreatic alpha and beta cells, and the co-stimulation of both insulin and glucagon secretion may account for the absence of any effect on graft outcomes in this study.

Co-activation of α2 and β2 receptors has been a poorly understood aspect of adrenergic receptor research. There is some evidence to support an acceleration of agonist-stimulated α2-receptor endocytosis under β2 co-activating conditions.^32^ Other research, however, suggests that co-activation of α2 and β2 receptors leads to desensitisation and downregulation of α2 receptors and results in a 67-fold reduction in the threshold concentration of adrenaline required for α2 downregulation.^33^ Given that noradrenaline was co-administered with dobutamine in 88% of cases, this hypothesis may provide an explanation for the lost attenuation of α2 receptor activity in the dobutamine subgroup.

Beta-cell rest may also be induced by the administration of exogenous insulin, and use of supplementary insulin therapy in both organ donors and pancreas and islet transplant recipients has been investigated.^34^ We have previously shown that use of insulin therapy to treat hyperglycaemia (glucose > 10mmol/L) in organ donors is associated with worse glycaemic control at 3 months after islet transplantation and also higher rates of isolated islet failure at 3 months following pancreas transplantation.^35,36^ It is conceivable that outcomes may have been poorer still in the cohort of donors experiencing hyperglycaemia had they not received exogenous insulin because of unattenuated beta-cell stress.

### Clinical and research implications

If these results can be replicated, then these data could make a valuable contribution to decision-making processes in donor pancreas selection. Existing donor pancreas risk prediction indices such as the Preprocurement Pancreas Suitability Score and Pancreas Donor Risk Index are poor predictors of outcomes.^25,26,37-39^ However, information on noradrenaline use has the potential to improve the selection of pancreases for transplantation. In vitro and in vivo mechanistic studies may help determine whether the observed relationships are causal. Identification of alternative methods of initiating beta-cell rest without instituting the concomitant side effects associated with VaDs could also be valuable.

Use of noradrenaline was considerably lower in donations after circulatory death (DCDs) compared with DBDs. This is most likely because, upon coning, DBDs experience a significant catecholamine and cytokine surge that is followed by a profound slump during which vasopressors and inotropes are commonly required to maintain adequate global perfusion. By their nature, DCDs do not experience these profound physiological changes prior to donation. During the assessment process for donation, DCDs are frequently more stable. Adverse outcomes in the DCD subgroup are more commonly associated with warm ischaemia arising between the withdrawal of life-sustaining therapies and the perfusion of cold preservation solutions.

### Strengths and weaknesses

This study has several strengths: it is the largest to assess relationships between VaDs and graft failure after pancreas transplantation; the entire UK experience of pancreas transplantation between 2004 and 2016 is reported; and several variables were accounted for that could confound relationships between exposures and outcome.

We acknowledged some limitations. First, the majority of donors received at least two VaDs, which means that there may be uncertainty around whether the data indicate that noradrenaline is ‘beneficial’ or that comparator drugs are ‘harmful’. However, for the mechanistic reasons outlined above, the former explanation may be more likely. We addressed the issue by also reporting data from the cohort of patients that received no other drugs, and by adjusting for all permutations of drug use and co-linearity in the multivariable model. Second, the study used routinely collected registry data with its inherent potential limitations: retrospective design, potential for variation in data quality and missing data. However, we were fortunate that the data quality in the study was relatively high with limited missing data. Third, even though the study was large, there were limited numbers of events in some subgroups. For example, important relationships between dobutamine use and graft failure may not have been adequately captured because of limited statistical power. Finally, the focus on graft failure limits the ability to identify relationships that could lead to the early identification of deteriorating graft function. The Igls criteria on defining outcomes in beta-cell replacement therapy addresses this to some extent, but because of the small numbers of transplants performed annually, accrual of a comprehensive dataset may take some time.^40^

## Conclusion

Noradrenaline use was associated with better graft survival after adjusting for potentially confounding donor and recipient variables – these benefits may be related to inhibition of pancreatic insulin secretion initiating beta-cell ‘rest’. Further research is required to validate these findings and to establish whether relationships are causal. Identification of alternative methods of initiating beta-cell rest could be valuable in improving graft outcomes.
